# From animal testing to model-informed drug development: building on ICH M15 and EMA initiatives to make new approach methodologies (NAMs) deliver

**DOI:** 10.3389/fphar.2026.1877947

**Published:** 2026-07-10

**Authors:** Jean-Michel Dogné, Médéa Locquet, Flora Musuamba, Sonja Beken

**Affiliations:** 1 Department of Pharmacy, Clinical Pharmacology and Toxicology Research Unit, Namur Research Institute for Life Sciences (NARILIS), Faculty of Medicine, University of Namur, Namur, Belgium; 2 Department of Biomedical Sciences, Clinical Pharmacology and Toxicology Research Unit, Namur Research Institute for Life Sciences (NARILIS), Faculty of Medicine, University of Namur, Namur, Belgium; 3 Belgian Federal Agency for Medicines and Health Products, Brussels, Belgium; 4 Faculty of Pharmaceutical Sciences, Université de Lubumbashi, Lubumbashi, Democratic Republic of Congo

**Keywords:** new approach methodologies, model-informed drug development, regulatory science, pharmacokinetic/pharmacodynamic modeling, artificial intelligence

## Abstract

The transition from animal-based experimentation toward New Approach Methodologies (NAMs) requires not only technological innovation but also regulatory frameworks that enable evidence-based decision-making. We discuss how the European Medicines Agency’s evolving strategy for regulatory acceptance of NAMs, combined with the ICH M15 guideline on Model-Informed Drug Development (MIDD), can support the integration of mechanistic pharmacokinetic/pharmacodynamic modeling, physiologically based pharmacokinetic models, quantitative systems pharmacology, and artificial intelligence-driven approaches into medicines development. We argue that NAMs and MIDD are complementary: NAMs generate human-relevant experimental and computational evidence, whereas MIDD provides a quantitative framework to integrate and evaluate this evidence for defined regulatory questions and contexts of use. We further propose that routine MIDD planning, context-of-use-based case studies, regulatory-grade models and data infrastructures, and shared regulatory expertise are needed to move NAMs toward more central regulatory evidence. Rather than implying an immediate replacement of animal studies, this approach could progressively support a more simulation-informed, human-relevant, and risk-based drug development paradigm.

## Introduction

The recent report by Burki in *The Lancet* reviews political pledges in the UK and USA to phase out animal experimentation and provides a timely overview of New Approach Methodologies (NAMs), from organ-on-chip technologies to AI-driven *in silico* toxicology (Burki, 2026). It concludes that NAMs will increasingly support and, in some areas, partially replace animal testing. However, the report only briefly mentions the European context. It does not explore how emerging regulatory frameworks, such as the ICH M15 guideline on Model-Informed Drug Development (MIDD) (European Medicines Agency EMA, 2026c) and the European Medicines Agency’s (EMA) strategy for regulatory acceptance of NAMs (European Medicines Agency EMA, 2026e), could translate these expectations into a coherent, qualitative, quantitative, and globally harmonized pathway away from animal-centered drug development.

Here, we argue that Europe is more advanced than often perceived in integrating the 3Rs (Replacement, Reduction, Refinement) into medicines regulation. We further argue that ICH M15 provides a robust framework to elevate NAMs from exploratory tools to primary decision-making evidence and that the next decisive step is to couple mechanistic pharmacokinetic and pharmacodynamic models with AI-enabled prediction within a clearly defined MIDD strategy, so that animal studies become scientifically exceptional rather than just procedurally standard (Medicines Agency - Heads of Medicines Agency, 2025; European Medicines Agency EMA, 2026b).

## Europe, European medicines regulatory network (EMRN) and EMA: from 3Rs principles to structured NAMs uptake

The European Union has long embedded the 3Rs principles of replacement, reduction, and refinement of animal use into its legal framework for scientific research and medicines testing, notably through Directive 2010/63/EU and its implementation in the pharmaceutical sector (EUR-Lex, 2010). Within the EMA, this commitment has evolved into a coordinated work plan, overseen since 2011 by a Joint CHMP/CVMP *ad hoc* Expert Group on 3Rs, which, in 2022, established the standing joint CHMP/CVMP 3Rs Working Party (3RsWP) (Empl et al., 2026). The 3RsWP Biennial Report 2023–2024 outlines the latest initiatives, demonstrating how the Agency has moved toward structured, transparent, and reviewable mechanisms to support the regulatory acceptance of NAMs as innovative tools that promote the 3Rs while maintaining or enhancing the scientific robustness necessary to protect human and animal health (Medicines Agency - Heads of Medicines Agency, 2025; European Medicines Agency EMA, 2026a).

Over the past years, EMA has established concrete mechanisms to promote regulatory acceptance of NAMs. A dedicated “regulatory acceptance of NAMs” pathway now offers multiple interaction formats, ranging from early advice via Innovation Task Force briefing meetings to product-specific and broad scientific advice. It can ultimately lead to formal regulatory acceptance through the qualification of novel methodologies and procedures. These interaction mechanisms are specifically designed to help concerned stakeholders (*e.g*., method developers, pharmaceutical companies, clinical research organizations) generate and present NAM-generated evidence in a way that can be evaluated for future regulatory decision-making (Medicines Agency - Heads of Medicines Agency, 2025; European Medicines Agency EMA, 2026a). Through voluntary data submissions, EMA can review NAM-derived data alongside traditional animal studies within marketing authorization procedures and in non-product-specific process contexts. This process aims to build confidence in NAM-generated evidence, refine or define the context of use, and identify the readiness and limitations of NAMs for regulatory acceptance. Ultimately, this can guide the development of context-of-use-based regulatory acceptance or qualification criteria. The guideline on the regulatory acceptance of 3Rs testing approaches is currently being updated and will include clear criteria for the acceptance of complex *in vitro* models applied for specific contexts of use (*for example*, cardiovascular safety pharmacology testing). The 3Rs Working Party identified multiple contexts of use where NAMs are already applicable and where future opportunities for NAM applications are imminent. Additionally, opportunities to reduce the use of non-human primates in the safety testing of human medicinal products have recently been published. This includes building on the existing flexibility within published guidelines to incorporate currently available 3Rs approaches and to highlight future opportunities. EMA led the establishment of the International Medicines Regulators Working Group on 3Rs to promote international dialogue on the application of the 3Rs in non-clinical and quality control testing and to work toward harmonized acceptance criteria (European Medicines Agency EMA, 2026d).

The EMA 3RsWP considers stakeholder engagement as key to progressively foster regulatory use and acceptance of methods such as complex *in vitro* models (*e.g*., organ-on-chip systems, organoids, microphysiological systems), computer modeling, and structured combinations thereof (European Medicines Agency EMA, 2026e; European Medicines Agency EMA, 2026b). The integration of knowledge in so-called weight-of-evidence approaches or integrated risk assessment (*e.g*., ICH S1B, S11, E14/S7B), combining human-based *in vitro* systems, *in silico* models, and, where still indispensable, judiciously designed *in vivo* studies, is considered a way forward to improve and innovate regulatory decision-making while supporting the 3Rs (European Medicines Agency EMA, 2026a). From this perspective, the European ecosystem is already structurally prepared to host a radical shift towards non-animal, model-informed development, as Burki’s report suggests (Burki, 2026; European Medicines Agency EMA, 2026c; European Medicines Agency EMA, 2026a).

As these approaches develop, important methodological and regulatory issues are being actively addressed. Efforts focus on improving the robustness and representativeness of the data used to train AI and *in silico* models, thereby strengthening their reliability and generalizability. In parallel, regulatory frameworks are clarifying the contexts of use in which NAMs can be considered sufficiently validated to serve as primary evidence, and how they should be combined with other sources of evidence.

### ICH M15: a global framework for model-informed evidence

NAMs and MIDD represent complementary but distinct concepts. NAMs are evidence-generating methodologies, such as human-based *in vitro* systems, organ-on-chip platforms, *in silico* tools, or AI-based prediction models, whereas MIDD provides a quantitative framework to integrate, evaluate, and communicate such evidence in relation to defined regulatory questions and contexts of use.

In this context, the ICH M15 guideline on general principles for MIDD offers a major opportunity for NAM innovation and regulatory decision-making (European Medicines Agency EMA, 2026c). M15 defines MIDD as the strategic use of computational modeling and simulation methods, including *in silico* approaches and encompassing empirical and mechanistic pharmacokinetic/pharmacodynamic models, physiologically based pharmacokinetic (PBPK) models, physiologically based biopharmaceutics models (PBBM), quantitative systems pharmacology, and emerging approaches such as agent-based models and AI/machine learning, to integrate nonclinical and clinical data, prior knowledge on mechanisms, and disease understanding to generate evidence for development and regulatory decisions.

The general principles of MIDD, per ICH M15, therefore include applying these methods each time they are used to describe, support, refine, or replace other types of evidence (e.g., those based on animal experiments or clinical trials) in the context of drug development.M15 emphasizes the principles guiding the proper use of MIDD, whether during the planning stage before data collection or at the data analysis stage once data are available. It also highlights the best way to report model assessment results and communicate with stakeholders. Risk-based analysis forms the core of the ICH M15 guideline. For each question and related context of use, the evaluation of model technicalities and suitability should be proportionate to the risk to the patient if the model fails, and should consider existing standards and alternatives.

Crucially, M15 applies to both current and emerging modeling methods and explicitly includes AI and machine learning in the MIDD toolbox (European Medicines Agency EMA, 2026c). The same conceptual framework can therefore be used to assess a classical population pharmacokinetic model and deep learning-based toxicity predictors, provided their contexts of use, performance, and limitations are transparently characterized (European Medicines Agency EMA, 2026c). For regulators such as EMA and EMRN, M15 offers a harmonized approach to move beyond *ad hoc* model use towards planned, systematic, and reviewable model-based evidence that can legitimately replace or reduce certain animal studies.

Interestingly, the recently approved final texts of the EU Pharma Package define non-clinical study as “a study or a test conducted *in vitro*, *ex vivo*, *in silico*, or *in chemico*, or *non-human in vivo* related to the investigation of the safety and efficacy of medicines. Such tests may include simple and complex human cell-based assays, microphysiological systems including organ-on-chip, computer modeling and other *in silico* methods, other non-human or human biology-based test methods, and animal-based tests.” (Union européenne, 2026).

### From NAMs as supplementary tools to NAMs as core evidence: integrating PBPK, PK/PD, and AI

Although animal models are widely used, they have limited predictive power for human outcomes. About 90% of drug candidates fail in clinical trials, mostly due to lack of effectiveness or unexpected toxicity despite promising preclinical results (Institutes of Health Advisory Committee to the Director and N, 2023). In the specific context of FDA-approved anticancer drugs, recent analyses also suggest only moderate agreement between animal and human safety outcomes, with success rates generally between 50% and 60%, depending on the endpoint and species. (Choi et al., 2026). These findings highlight fundamental limits of animal testing and support the development of more human-relevant, model-based approaches that combine mechanistic insights with advanced *in vitro* and *in silico* methods.

Burki already rightly emphasizes organ-on-chip platforms, advanced *in vitro* models, and AI-based toxicity prediction as key NAMs driving the political goal of “make animal studies the exception rather than the norm.” (Burki, 2026) However, MIDD was not identified as a central contributor to the current shift toward alternatives to animal testing. The current regulatory framework offers an opportunity to incorporate NAM-generated data into a quantitative, pharmacology-driven framework, as described in ICH M15, to address key regulatory questions such as dose selection, dose-response relationships, population extrapolation, and safety margins (Medicines Agency - Heads of Medicines Agency, 2025; European Medicines Agency EMA, 2026b; Lin and Chou, 2022). Mechanistic pharmacokinetic and pharmacodynamic models, including PBPK and QSP models, are recognized by EMA as part of the construct of NAMs capable of supporting various applications, including dose justification for early and late-phase clinical trials, bridging across formulations and modes of administration, drug-drug interaction assessment, and extrapolation of efficacy and/or safety to special populations (*e.g*., children, pregnant women, subpopulations underrepresented in clinical studies) (European Medicines Agency EMA, 2026e; European Medicines Agency EMA, 2026b). Using the principles outlined in M15, such models can be planned prospectively, evaluated through a risk-based approach, and used to simulate clinical scenarios that would otherwise require extensive animal data (European Medicines Agency EMA, 2026c). The guideline explicitly encourages early integration of MIDD into the overall development plan, with alignment between modeling activities, decision points, and study designs.

A key next step is to systematically augment these (mechanistic) models with AI tools and to combine them with other relevant methodologies and data to address interconnected regulatory questions. Machine learning has already demonstrated good performance in predicting certain toxicological endpoints and estimating pharmacokinetic parameters from molecular structure and limited experimental data (Lin and Chou, 2022). Recent work indicates that AI-enabled toxicology models trained on large repositories can quickly screen new compounds and identify high-risk candidates before they enter animal testing. This approach reduces the number of *in vivo* studies needed and ensures that animal use is focused on compounds for which model-based uncertainty remains high (Lin and Chou, 2022). Rather than functioning as standalone “black-box” predictors, AI components can refine parameter estimation by identifying relevant covariates and updating prior knowledge within a transparent, model-based framework. This paradigm aligns with the core principles of MIDD, in which the objective is not only to predict outcomes but also to generate mechanistically based, quantitatively reliable evidence that supports causal reasoning across regulatory questions.

Within an MIDD strategy, hybrid designs can be developed that combine mechanistically interpretable PBPK or disease progression models with AI components for parameter estimation, covariate discovery, or surrogate biomarker prediction (Lin and Chou, 2022). Under M15, these models would be accompanied by a rigorous assessment of model risk, comprehensive documentation of training data and validation performance, and clear articulation of the regulatory decisions they aim to support (*e.g*., justifying the omission of a dedicated non-rodent pharmacokinetic study or the use of human-relevant *in vitro* systems instead of certain toxicity assays) (European Medicines Agency EMA, 2026c; European Medicines Agency EMA, 2026e). Beyond its predictive role, the integration of AI within an MIDD framework should primarily be seen as a way to strengthen causal inference and reduce uncertainty in regulatory decision-making. Recent work illustrates how machine learning can be combined with mechanistic models such as PBPK or disease progression models to improve human translatability while preserving biological interpretability (Marshall et al., 2023). Hybrid approaches that couple mechanistic modeling with Bayesian statistical frameworks explicitly characterize uncertainty and inter-individual variability, enabling more robust extrapolation from preclinical to clinical settings (Marshall et al., 2023).

Nevertheless, the transition toward simulation-centered development should not be interpreted as implying that NAMs and MIDD approaches can already address all regulatory concerns. Important scientific and technical limitations remain, particularly for complex toxicity endpoints involving long-term, multi-organ, immune-mediated, or developmental mechanisms. PBPK models may vary across platforms, assumptions, input data, and levels of physiological detail, while QSP models remain difficult to validate in regulatory contexts because they often describe complex biological networks with limited external confirmation. AI-based toxicity predictors may also be affected by overfitting, limited transparency, poor generalizability, and non-representative training datasets. For these reasons, the use of NAMs and MIDD as regulatory evidence should rely on clearly defined contexts of use, transparent reporting, sensitivity and uncertainty analyses, reproducibility, standardization, and independent validation, particularly for high-impact regulatory decisions.

### A European opportunity: building simulation-centric, human-relevant development pathways

If the political goal is to “end animal testing wherever possible,” the regulatory question is not only which technologies qualify as NAMs, but how to design development programs that rely on human-relevant data and model-based simulations as the core evidence. Europe is uniquely positioned to lead this transition for several reasons.

First, EMA’s 3Rs activities, dedicated structures to support innovation while fostering the 3Rs and thereby ethical use of animals, and structured NAMs interaction pathways already provide an institutional home for developers wishing to discuss new approach methodologies and *in silico* approaches early in development (European Medicines Agency EMA, 2026e; Medicines Agency - Heads of Medicines Agency, 2025; EUR-Lex, 2010; European Medicines Agency EMA, 2026a).

Second, the implementation of ICH M15 within the European regulatory framework will provide a harmonized basis for planning and evaluating model-based evidence, including AI-enabled methods, across ICH regions (ICH M15 guideline on general; European Medicines Agency EMA, 2026a).

Third, complementary EU-level initiatives, such as horizon-scanning exercises on NAMs, the European Research Action on NAMs in biomedical research and regulatory testing of pharmaceuticals, and the EC roadmap to phase out animal testing in chemicals regulation, show a political willingness to invest in validation/qualification, standardization, and capacity-building for NAMs (European Medicines Agency EMA, 2026b; European Commission, 2023).

Beyond individual case studies, Europe should invest in sustained ‘regulatory-grade’ infrastructure for MIDD-enabled NAMs. This includes curated, shareable model libraries, traceable datasets, and common documentation standards that allow both regulators and sponsors to understand how models were built, calibrated, and updated over time. Such infrastructure would lower the entry barrier for smaller developers, support independent re-use and stress-testing of models, and help ensure that simulation-based evidence is not confined to a few highly specialized centers.

To take advantage of this opportunity, we suggest several recommendations.

First, make the MIDD strategy routine for innovative products. For new entities, especially biologics and advanced therapies, regulators should encourage or require a MIDD strategic plan that explicitly describes how *in vitro*, *in silico*, and *in vivo* data will be integrated, with a presumption in favor of NAMs whenever scientifically and plausibly supported by prior mechanistic knowledge and available data (European Medicines Agency EMA, 2026c; Medicines Agency - Heads of Medicines Agency, 2025).

Second, identify the key question and the regulatory settings where NAMs using these MIDD approaches can replace, reduce, or improve specific animal studies. Building on EMA’s NAMs/3Rs regulatory acceptance framework, case studies should be developed that, for example, incorporate PBPK, human-based complex *in vitro* data, and AI predictions to serve as primary evidence for specific regulatory questions (*e.g*., in pharmacokinetic bridging settings), rather than just supporting information.

Third, invest in “regulatory-grade” model and data infrastructures. Public-private consortia should develop shared, transparent, and well-curated model libraries and data repositories that meet M15 documentation and validation standards, thereby lowering barriers for regulators and sponsors to rely on simulation-based evidence.

With its 3Rs principles, the EMA and its 3RsWP clearly aim to promote the 3Rs while improving human relevance and evidence, making regulatory science more predictive. This will involve integrating AI and other data-driven methods into mechanistic pharmacology and toxicology, integrating human-derived *in vitro* data (*e.g*., microphysiological systems such as organ-on-chip), and thoroughly assessing uncertainty, all without replacing one opaque black box (animal model) with another (an uninterpretable algorithm) (European Medicines Agency EMA, 2026c; European Medicines Agency EMA, 2026a; Lin and Chou, 2022).

Nevertheless, having regulatory frameworks formally established does not automatically translate into routine implementation. Wider use of NAMs and MIDD as regulatory evidence is still limited by the need for regulatory-grade validation data, consistent model quality and documentation, access to curated and representative datasets, and shared expertise across developers, assessors, and regulators. Adoption may also be slowed by economic constraints and by the familiarity of established animal-based pathways. Operationalization, therefore, requires not only guidance but also capacity building, resources, transparent case studies, harmonized standards, and practical experience with model-informed evidence in specific regulatory decisions.

To fully realize this opportunity, investment in human expertise is as important as investment in technology. Regulators, assessors, and developers will need training in quantitative pharmacology, data science, and AI, as well as practical experience in interpreting complex model outputs and their uncertainties. Building this shared expertise and a culture of transparent, question-driven modeling is essential if simulation-centric, human-relevant development pathways are to become the norm rather than the exception.

The success of this transition should also be evaluated through measurable indicators. These could include the proportion of regulatory submissions in which NAMs or MIDD approaches are prospectively used to address defined questions, the number of animal studies reduced, refined, or replaced within accepted contexts of use, the concordance between model-based predictions and observed clinical or post-authorization outcomes, and the reproducibility of model results across platforms or independent datasets. Additional indicators may include qualification of specific NAMs for regulatory use, reductions in development uncertainty, and the extent to which model-informed evidence contributes to dose selection, population extrapolation, or safety margin assessment.

## Conclusion

The report by Burki rightly highlights the enthusiasm around NAMs and the political momentum to reduce animal use (Burki, 2026). However, MIDD was not mentioned as a key player in the current shift toward leveraging alternatives to animal testing. To make this transition scientifically credible and sustainable, regulation must shift from an animal-centric default to a simulation-centric paradigm based on human biology. In Europe, EMA’s long-standing commitment to the 3Rs and its emerging framework for regulatory acceptance of NAMs, combined with the global harmonization offered, for example, by ICH M15, provides exactly the foundation needed to enable this shift. The challenge now is to operationalize these frameworks through model-informed programs that deliver quantitative, AI-enhanced predictions and to ensure that animal studies are scientifically justified as true exceptions.

## Data Availability

The original contributions presented in the study are included in the article/supplementary material, further inquiries can be directed to the corresponding author.
